# The spliceosome factor *sart3* regulates hematopoietic stem/progenitor cell development in zebrafish through the p53 pathway

**DOI:** 10.1038/s41419-021-04215-4

**Published:** 2021-10-05

**Authors:** Yan Zhao, Mei Wu, Jing Li, Ping Meng, Jiakui Chen, Zhibin Huang, Jin Xu, Zilong Wen, Wenqing Zhang, Yiyue Zhang

**Affiliations:** 1 Division of Cell, Developmental and Integrative Biology, School of Medicine, South China University of Technology, Guangzhou, 510006 P.R. China; 2 School of Biology and Biological Engineering, South China University of Technology, Guangzhou, 510006 P.R. China; 3 Department of Developmental Biology, School of Basic Medical Sciences, Southern Medical University, Guangzhou, 510515 P.R. China; 4 State Key Laboratory of Molecular Neuroscience, Division of Life Science, The Hong Kong University of Science and Technology, Hong Kong, P.R. China

**Keywords:** Haematopoiesis, Haematopoietic stem cells

## Abstract

Hematopoietic stem cells (HSCs) possess the potential for self-renew and the capacity, throughout life, to differentiate into all blood cell lineages. Yet, the mechanistic basis for HSC development remains largely unknown. In this study, we characterized a zebrafish *smu471* mutant with hematopoietic stem/progenitor cell (HSPC) defects and found that *sart3* was the causative gene. RNA expression profiling of the *sart3*^*smu471*^ mutant revealed spliceosome and p53 signaling pathway to be the most significantly enriched pathways in the *sart3*^*smu471*^ mutant. Knock down of *p53* rescued HSPC development in the *sart3*^*smu471*^ mutant. Interestingly, the p53 inhibitor, *mdm4*, had undergone an alternative splicing event in the mutant. Restoration of *mdm4* partially rescued HSPC deficiency. Thus, our data suggest that HSPC proliferation and maintenance require *sart3* to ensure the correct splicing and expression of *mdm4*, so that the p53 pathway is properly inhibited to prevent definitive hematopoiesis failure. This study expands our knowledge of the regulatory mechanisms that impact HSPC development and sheds light on the mechanistic basis and potential therapeutic use of *sart3* in spliceosome*-mdm4-*p53 related disorders.

## Introduction

Hematopoiesis is tightly regulated by multidimensional pathways [1–3], with dysregulation resulting in anemia, leukemia, and other hematological diseases [4, 5]. Blood development in vertebrates is comprised of primitive and definitive waves [6]. In mammals, the first hematopoietic wave takes place in the yolk sac and gives rise to primitive erythrocytes and macrophages that support tissue oxygenation and immune defense during early embryonic development [1, 7]. Primitive hematopoiesis is transitory and followed by definitive hematopoiesis, which includes yolk sac-derived erythroid-myeloid progenitors. These progenitors contribute to the intermediate wave [8], with later hematopoietic stem cells (HSCs) contributing to hematopoiesis throughout life [9]. Definitive HSCs, which can give rise to all blood lineages, originate from an endothelial-to-hematopoietic transition process within the aorta-gonad mesonephros (AGM) region [10, 11]. These cells migrate to the fetal liver and colonize bone marrow (BM), where they maintain adult hematopoiesis [12, 13]. Similarly, zebrafish hematopoiesis is also comprised of a first primitive wave, a transient erythroid-myeloid progenitor wave, and a definitive HSC contributed wave [14]. HSCs emerge from the ventral wall of the dorsal aorta (VDA, equivalent to the mammalian AGM) and enter the circulation after initiation of the endothelial-to-hematopoietic transition process [15, 16]. The cells colonize the caudal hematopoietic tissue (CHT, equivalent to the mammalian fetal liver) [17, 18], then migrate to adult hematopoietic organs, which include the thymus as well as kidney marrow (equivalent to the mammalian BM) [1]. As HSCs are the apex of the blood hierarchy and have the potential to self-renew and differentiate into all blood cell lineages [19, 20], analysis of HSC biology is essential for a better understanding of hematopoiesis and the treatment of blood disorders.

In the past few decades, spliceosome genes most frequently involved in hematopoiesis and leukemogenesis have been identified including; *U2AF1*, *SF3B1*, and *SRSF2* [21–23]. Mice with the U2AF1 (S34F) mutation exhibit a defect in hematopoiesis as well as an alteration in pre-mRNA splicing in BM progenitors [24]. Mice bearing the *Sf3b1*^K700E^ mutation exhibit decreased numbers of mature erythrocytes and long-term HSC expansion [25]. Mice bearing the *Srsf2*P95H mutation have key hematopoietic regulator splicing mistakes as well as impaired hematopoietic differentiation [26]. These mutations occur in myelodysplastic syndrome, chronic myelomonocytic leukemia, and acute myeloid leukemia, which can also be used as prognostic biomarkers of disease [22, 23]. Moreover, several studies have shown these spliceosome genes (such as *SF3B1*) to be potential antitumor targets [25, 27–29]. Therefore, exploring novel spliceosome genes and their role in hematopoiesis will benefit the development of new therapeutic strategies for treatment of blood diseases.

Squamous cell carcinoma antigen recognized by T cells 3 (SART3), also known as p110/p110^nrb^ [30] or TIP110 [31], is a homolog of yeast spliceosome recycling factor Prp24 [32] and an RNA-binding protein [30]. Similar to yeast Prp24, mammalian SART3 likely reassembles U4/U6 small nuclear ribonucleoproteins (snRNPs), affecting pre-mRNA splicing by binding to U6 snRNP [32–34]. Moreover, SART3 is multifunctional with involvement in gene regulation [35–37], cancer immunology [38–40], stem cell pluripotency maintenance [41, 42], embryonic development [43], and hematopoiesis [36, 43, 44]. During BM hematopoiesis, *Sart3* haplo-insufficient mice exhibit decreased numbers of hematopoietic progenitor cells (HPCs) and *SART3* transgenic mice exhibit increased HPCs, likely due to cell cycle regulation mediated by CMYC [36]. Trede et al. reported a zebrafish *sart3* mutant with a lymphoid cell deficiency but normal levels of primitive erythrocytes [43], supporting *sart3* involvement in hematopoiesis. However, the role of SART3, in early hematopoietic stem/progenitor cell (HSPC) development, is largely undefined.

In this study, we characterized a zebrafish *sart3*^*smu471*^ mutation, which resulted in a deficiency of HSPC development largely due to activation of the p53 pathway. Moreover, we found that *sart3* mutation-induced global splicing changes that included alternative splicing (AS) of *mdm4*, one key p53 negative regulator, which triggered p53 activation, impacting HSPC proliferation and maintenance. Taken together, this study identifies a novel role for splicing factor and *sart3* in regulation of early HSPC development.

## Results

### The *smu471* mutant is defective in HSPC development

**Fig. 1:**
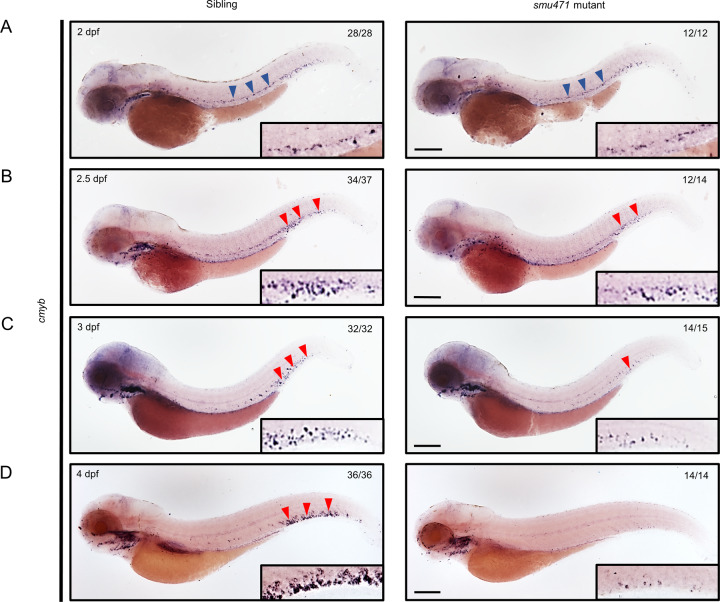
Decreased HSPCs in the CHT region of zebrafish *smu471* mutants. **A–D***cmyb* WISH identified HSPCs from 2 to 4 dpf in *smu471* mutants (right) compared with their siblings (left). **A** *cmyb*^*+*^ signals were not altered at 2 dpf in the VDA region (blue arrowheads indicate signals in the region). **B** In the CHT region, *cmyb*^*+*^ signals were decreased slightly at 2.5 dpf in *smu471* mutants (red arrowheads indicate signals in the region). **C**, **D** Severely decreased *cmyb*^*+*^ signals in the CHT region at 3 dpf (**C**) with a further reduction at 4 dpf (**D**) in *smu471* mutants. The boxed regions in the lower right corner are magnifications. Scale bars: 200 µm.

To explore novel genes and pathways involved in definitive hematopoiesis, we previously performed an N-ethyl-N-nitrosourea-based large-scale forward genetics screening to identify mutants with hematopoietic defects [45]. One of the mutants, *smu471*, did not display the erythroid marker (*βe1*-globin) or the lymphoid marker (*rag1*) at 5 days post fertilization (dpf), but the HSPC marker (*cmyb*) was observed at 36 h post fertilization (hpf) [45] (Fig. S1). Next, primitive hematopoiesis was examined. The erythrocyte progenitor marker *gata1* (Fig. S2A) and the myeloid progenitor marker *pu.1* (Fig. S2B, C) were intact, suggesting that primitive hematopoiesis was unaffected by the mutation. Since definitive HSPCs can differentiate into all blood lineages, both 5-dpf erythroid and lymphoid disruption in *smu471* prompted us to speculate that HSPCs were likely defective in the mutant. HSPCs are generated from the VDA ~30 hpf [15, 16] and then migrate, differentiate, and expand in the CHT [1, 17, 18]. We therefore examined the HSPCs in the respective regions for HSPC marker (*cmyb*, *runx1*, and *gata2b*) expression at 36 hpf and 2 dpf in the *smu471* mutant. The results showed that *cmyb*- (Fig. 1A and Fig. S1C), *runx1*- (Fig. S3A, B), and *gata2b*- (Fig. S3C, D) marked HSPCs in the VDA region, were similar to siblings of the *smu471* mutant. Notably, *cmyb* expression was detected at 2.5 dpf (Fig. 1B), 3 dpf (Fig. 1C), and 4 dpf (Fig. 1D) in the CHT region but was obviously decreased in the *smu471* mutant from 2.5 dpf onward. Since the *Tg(cd41:eGFP)* transgenic fish line labels developing HSPCs in the VDA and CHT region, with *eGFP*^low^ cells as HSPCs and *eGFP*^high^ cells as thrombocytes [46], we monitored VDA and CHT region HSPC number using the inter-crossed progenies of the *sart3*^*+/smu471*^;*Tg(cd41:eGFP)*^*+/+*^ transgenic zebrafish. The results showed that the *cd41:eGFP*^low^ cells were intact at 2 dpf in the VDA and CHT region, but were decreased from 2.5 to 4 dpf in the CHT region in *smu471* mutants compared with their siblings (Fig. S4A–G), which were consistent with the WISH expression of *runx1*, *gata2b*, and *cmyb*. Moreover, we found the proportion of *cd41:eGFP*^low^ cell population at 4 dpf in *smu471* mutants was markedly decreased compared with their siblings using Fluorescence-activated cell sorting (FACS) (Fig. S4H, I). These results suggest that HSPCs arise normally from the VDA region whereas CHT localized HSPCs are affected in the *smu471* mutant.

### *Sart3* is the causative gene of the *smu471* mutation

**Fig. 2:**
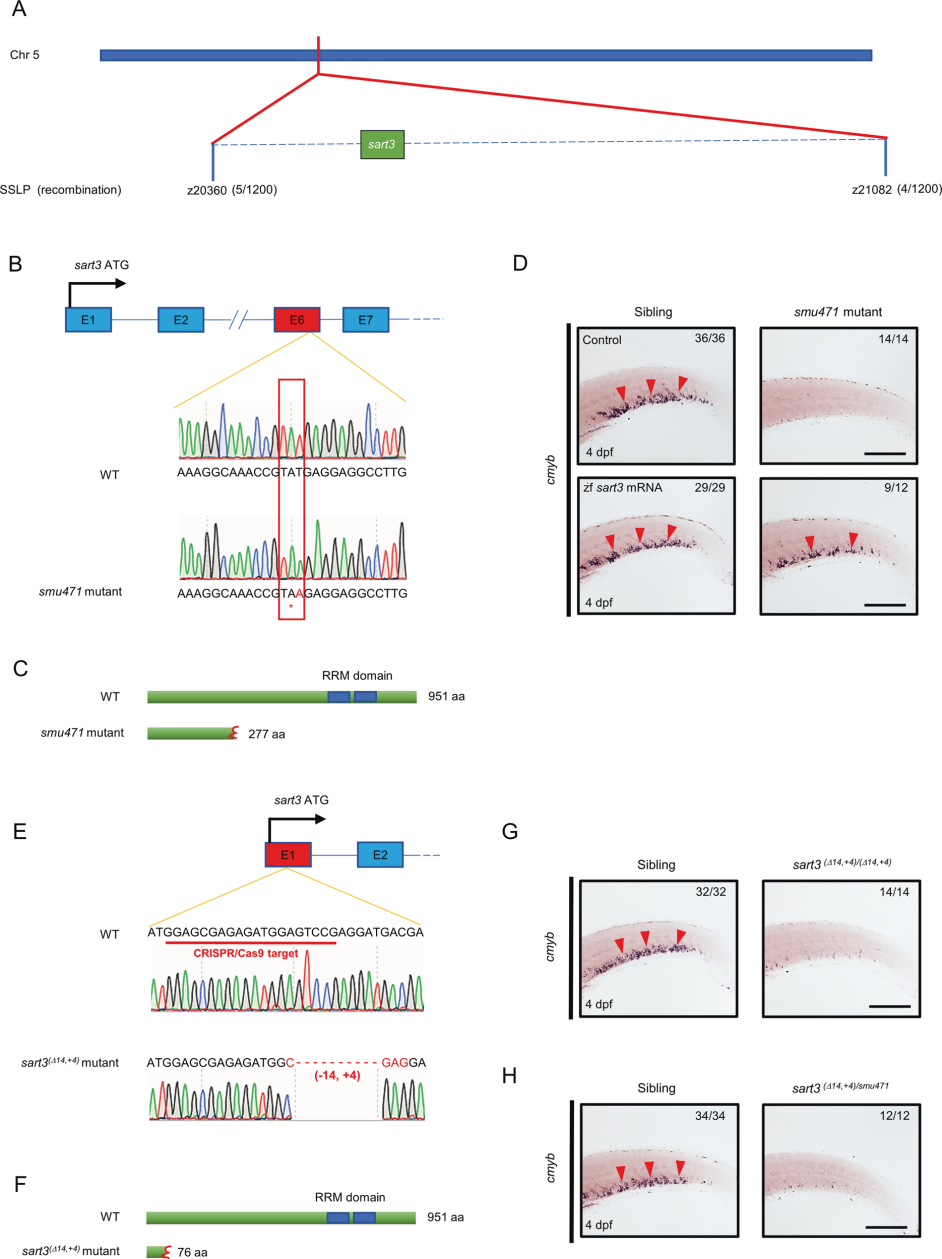
*sart3* is the causative gene of the zebrafish *smu471* mutation. **A** Genetic map of the candidate region on chromosome 5 (Chr 5) located between two SSLP markers, z20360 (5 recombinants in 1200 *smu471* mutants) and z21082 (4 recombinants in 1200 *smu471* mutants). There are twenty candidate genes in this mapped region. **B**, **C** Sequencing of the candidate gene cDNAs showed a transition of T to A at *sart3* exon 6, which predicted a stop codon (**B**) and truncated Sart3 protein (**C**). **D** Rescue assay. Zebrafish *sart3* mRNA (zf *sart3* mRNA) overexpression restored defective *cmyb* expression in *smu471* mutants. **E** A 14-bp deletion and 4-bp addition in *sart3* exon 1 were identified in CRISPR/Cas9 generated mutants. Sequence underlined in red indicates the CRISPR/Cas9 target in *sart3*. **F** Premature Sart3 protein generated by the *sart3*^*(Δ14,+4)*^ mutant. **G** *cmyb*^*+*^ signals decreased in 4 dpf *sart3*^*(Δ14,+4)*^ mutants. **H** *cmyb* + signals decreased in the 4 dpf bi-allelic *sart3*^*(Δ14,+4)*/*smu471*^ mutants. The red arrowheads indicate the *cmyb* signals. Scale bars: 200 µm.

To identify the causative gene responsible for the defective HSPCs of the *smu471* mutant, we performed positional cloning. We located the mutant gene on chromosome 5 by initial mapping. Next, we used fine mapping to identify the candidate interval between two simple sequence length polymorphism (SSLP) markers: z20360 and z21082, which contained 20 candidate genes (Fig. 2A). By sequencing the cDNA of these candidate genes, we found a T to A base substitution at *sart3* exon 6, which encoded a stop codon (Fig. 2B). The nonsense mutation predicted a truncated Sart3 protein lacking its RNA recognition motif (RRM) domain (Fig. 2C).

To confirm *sart3* as the causative gene of the *smu471* mutant, we performed a rescue experiment by overexpressing zebrafish *sart3* mRNA in *smu471* mutant embryos. Zebrafish *sart3* mRNA recovered *cmyb* and *rag1* expression of the *smu471* mutant (Fig. 2D, S5A). Furthermore, human *SART3* mRNA also rescued the hematopoietic defect of the *smu471* mutant (Fig. S5B), suggesting functional conservation across species. Notably, we generated another *sart3* mutant zebrafish by CRISPR/Cas9, which had 14-base pairs deleted and 4-base pairs added in exon 1, identified as *sart3*^*(Δ14,+4)*^ (Fig. 2E). The mutant predictably generated a premature Sart3 protein (Fig. 2F) that exhibited the same phenotype as the *smu471* mutant (Fig. 2G). Moreover, we outcrossed heterozygous *smu471* with heterozygous *sart3*^*+/(Δ14,+4)*^ producing a bi-allelic mutant *sart3*^*(Δ14,+4)/smu471*^, which had a similar HSPC defect as the *smu471* mutant (Fig. 2H). Taken together, these data indicate that *sart3* is the causative gene of the *smu471* mutant zebrafish (named *sart3*^*smu471*^ hereafter).

### HSPCs undergo arrested proliferation and increased apoptosis in the *sart3*^*smu471*^ mutant

Based on the published time-series RNA-seq data of the zebrafish CHT region [47] (http://www.picb.ac.cn/hanlab/ichtatlas/Home/), we found the dynamic expression of *sart3* to gradually increase with HSPC developmental stage. The *sart3* expression in HSPC was low at HSPC initiation (28–36 hpf) and gradually increased from 52 hpf to 4 dpf (Fig. S6), suggesting a role for *sart3* in HSPC expansion and maintenance within the CHT region.

**Fig. 3:**
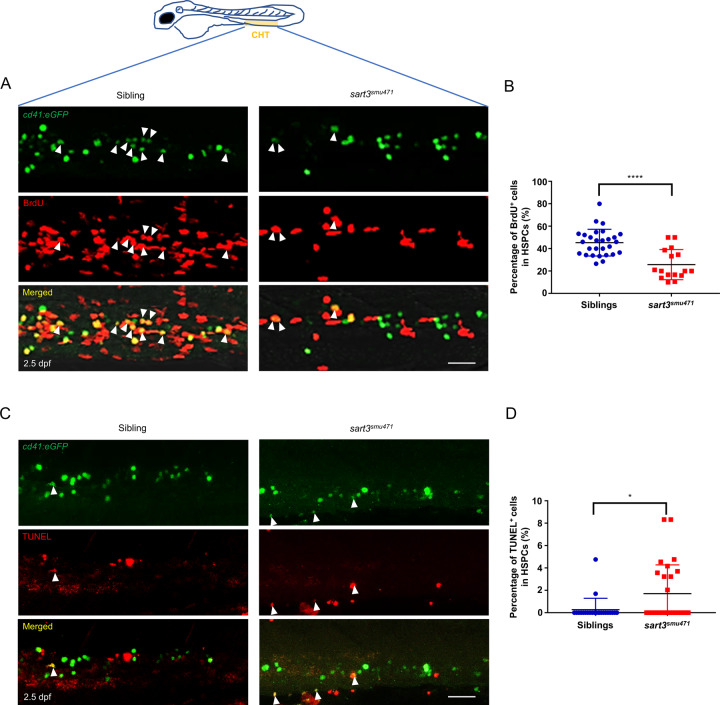
Decreased HSPC proliferation and increased apoptosis in *sart3*^*smu471*^ mutants. **A**, **B** Decreased HSPC proliferation in *sart3*^*smu471*^ mutants. **A** The BrdU incorporation assay. The white arrowheads indicate proliferating HSPCs (*cd41*:*eGFP*^low^; BrdU double positive cells). Green indicates eGFP, red indicates BrdU. **B** Statistical analysis of the percentage of CHT localized *cd41*^low^ HSPCs that incorporate BrdU (Student’s *t*-test, means ± SD; siblings, *n* = 28; *sart3*^*smu471*^ mutants, *n* = 16; ****P < 0.0001). **C**, **D** Increased HSPC death in *sart3*^*smu471*^ mutants. **C** The TUNEL assay. The white arrowheads indicate HSPCs undergoing cell death (*cd41*:*eGFP*^low^; TUNEL double positive cells). Green indicates eGFP, red indicates TUNEL. **D** Statistical analysis of the percentage of CHT localized *cd41*^low^ HSPCs labeled by TUNEL (Mann–Whitney *U* test, means ± SD; siblings, *n* = 24; *sart3*^*smu471*^ mutants, *n* = 27; *P < 0.05). Scale bars: 50 µm.

We thereby examined cell proliferation and cell death in *sart3*^*smu471*^ mutant HSPCs in the CHT region. We monitored CHT region HSPC status using 2.5-dpf inter-crossed progenies of the *sart3*^*+/smu471*^;*Tg(cd41:eGFP)*^*+/+*^ transgenic zebrafish. Measurement of cell proliferation by the bromodeoxyuridine (BrdU) incorporation assay found the percentage of BrdU labeled *cd41:eGFP*^low^ HSPCs (*eGFP*^low^; BrdU double positive cells) to be significantly reduced in *sart3*^*smu471*^ mutants compared to their sibling controls (Fig. 3A, B), suggesting that HSPC proliferation was attenuated by the *sart3* mutation. We then monitored cell death in the *sart3*^*smu471*^ mutant by terminal deoxynucleotidyl transferase (TdT)-mediated dUTP nick-end labeling (TUNEL) assay. Results showed the percentage of *cd41:eGFP*^low^ and TUNEL double positive cells to be significantly increased (Fig. 3C, D), suggesting that HSPC maintenance was also affected by the *sart3* mutation. Taken together, the above data suggest that the HSPC deficiency in *sart3*^*smu471*^ mutant is due to both decreased cell proliferation and increased cell death.

### The HSPC defect in the *sart3*^*smu471*^ mutant is p53 dependent

**Fig. 4:**
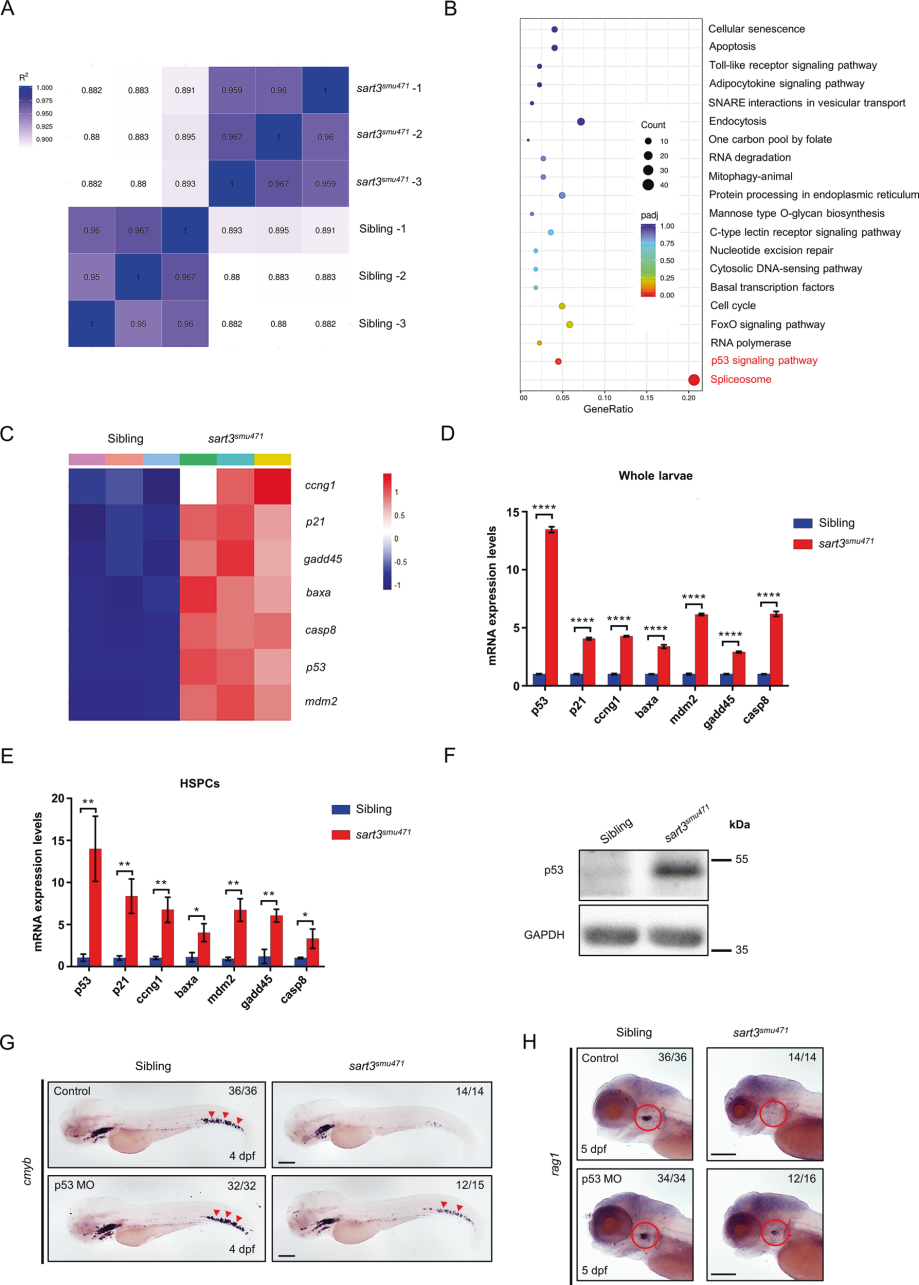
Reductions in HSPCs are p53 dependent in *sart3*^*smu471*^ mutants. **A** Distance heatmap analysis of WT siblings and *sart3*^*smu471*^ mutants. The color scale is shown on the left. The deeper the blue block the less different the samples are. **B** Spliceosome and p53 signaling pathway enrichment in *sart3*^*smu471*^ mutants by KEGG analysis of the RNA-seq data. **C** Heatmap demonstrating upregulation of p53 pathway genes in *sart3*^*smu471*^ mutants (data was normalized by Z-score, Z-score was shown on the right). **D**, **E** RT-qPCR validation of the RNA-seq data. Upregulation of *p53* and its downstream target genes *p21, ccng1, baxa, mdm2, gadd45*, and *casp8* in *sart3*^*smu471*^ whole larvae (**D**) and HSPCs (**E**) at 4 dpf (Student’s *t*-test, means ± SD, *n* ≥ 15 per group for whole larvae, *n* ≥ 2 × 10^4^ per group for HSPCs, ns: not significant, *P < 0.05, **P < 0.01, ****P < 0.0001). **F** Increased p53 protein in 4 dpf *sart3*^*smu471*^ mutants by western blot analysis. GAPDH was used as the loading control. **G**, **H** Phenotype restoration in *p53* knockdown *sart3*^*smu471*^ mutants. Red arrowheads indicate WISH signals of *cmyb* at 4 dpf in the CHT region (**G**) and red circles indicate *rag1* signals at 5 dpf in the thymus region (**H**). Scale bars: 200 µm.

Previous analysis has shown *SART3* to control *CMYC* and *GATA2* expression during mouse BM hematopoiesis [36], which could partially explain adult HPC cell cycle defects caused by a *Sart3* deficiency. Yet, how *Sart3* regulates embryonic HSPC proliferation and maintenance is still unclear. To analyze the molecular mechanism, we performed RNA sequencing (RNA-seq) analysis to assess *Sart3* regulation of HSPC expansion. Since the HSPC defect was evident at 4 dpf, RNA from *sart3*^*smu471*^ mutants and their wild-type siblings at this stage was collected for sequencing. Distance heatmap analysis of siblings and *sart3*^*smu471*^ mutants showed correlations for each biological replicate. Siblings and *sart3*^*smu471*^ mutants could be divided into two clusters (Fig. 4A). Through Kyoto Encyclopedia of Genes and Genomes (KEGG) pathway analysis, we found that the ‘spliceosome’ and the ‘p53 signaling pathway’ were the most significantly enriched pathways in *sart3*^*smu471*^ mutants compared with their siblings (Fig. 4B). Specifically, we found a series of genes (*ccng1*, *p21*, *gadd45*, *baxa, casp8*, *p53*, and *mdm2*) that were upregulated by *sart3* mutation (Fig. 4C), all of which were related to *p53* involved cell cycle or apoptosis pathways. To validate the RNA-seq data, we evaluated gene expression by real-time quantitative polymerase chain reaction (RT-qPCR). Consistent with the RNA-seq data, expression of *p53*-related genes was significantly elevated in *sart3*^*smu471*^ mutants compared with siblings (Fig. 4D), confirming activation of the p53 pathway in the *sart3*^*smu471*^ mutants. To determine whether p53 pathway activation occurred in mutant HSPCs, we examined *p53*-related upregulated gene expression in sorted *cd41:eGFP*^low^ labeled HSPCs. We found the expression of these genes to be significantly upregulated in the HSPCs of *sart3*^*smu471*^ mutants compared with their siblings (Fig. 4E), indicating the HSPC defect to be associated with p53 pathway activation.

We also found the level of p53 protein to be increased in *sart3*^*smu471*^ mutants compared with their siblings (Fig. 4F), confirming the p53 activation by *sart3* mutation. It is reported that p53 pathway activation is associated with cell cycle arrest and apoptosis [48]. Furthermore, our results suggest that *sart3* may regulate HSPC proliferation and apoptosis through activation of the p53 pathway. To assess this possibility, we outcrossed a *sart3* heterozygote with a *p53*^*M214K*^ mutant in which p53 function was reported to be abrogated [49]. Unfortunately, we failed to obtain a double mutant because of the short distance between *sart3* and *p53* genes on chromosome 5. As an alternative, we knocked down p53 in *sart3*^*smu471*^ mutants by *p53* morpholino oligonucleotide (MO). As expected, downregulation of p53 rescued the HSPC (Fig. 4G) and lymphoid defect (Fig. 4H) in *sart3*^*smu471*^ mutants. Collectively, these results demonstrate that *sart3* regulates HSPC development through the p53 pathway.

### *sart3* mutation induces AS of *mdm4*

**Fig. 5:**
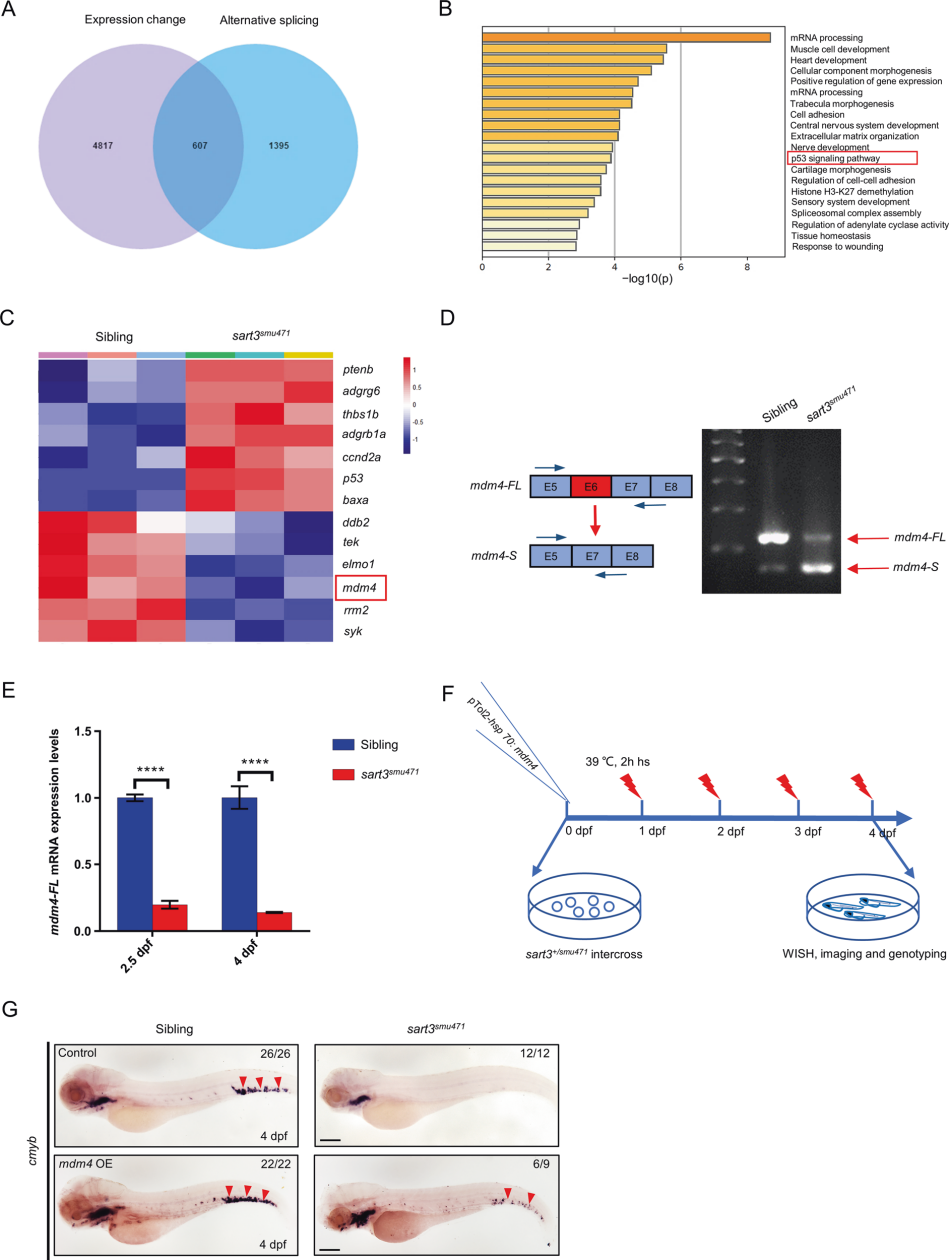
The *sart3* mutation triggers alternative splicing of *mdm4* as well as the activation of the p53 pathway. **A** Overlap of gene expression change (padj < 0.05) with alternative splicing (padj < 0.05) in *sart3*^*smu471*^ mutants from RNA-seq data. The overlapping number is 607. **B** p53 signaling pathway enrichment by GO term enrichment and KEGG pathway analyses from the 607 overlapped genes. The horizontal axis represents the significance, −log10(p). **C** Heatmap of p53 signaling pathway-related gene changes exhibited in (**B**) (data was normalized by Z-score, Z-score was shown on the right). The p53 negative regulator, *mdm4*, is boxed. **D** *mdm*4 alternative splicing and primers designed for PCR detection of exon 6 skipping. The *mdm4* full-length transcript (*mdm4-FL*) decreases and truncated *mdm4* transcript (*mdm4-S*) increase in *sart3*^*smu471*^ mutants. **E** Decreased expression of *mdm4-FL* by RT-qPCR at 2.5–4 dpf *sart3*^*smu471*^ mutants (Student’s *t*-test, means ± SD, *n* ≥ 15 per group, ****P < 0.0001). **F** The procedure for the *mdm4* overexpression rescue of reduced HSPCs in *sart3*^*smu471*^ mutants. hs: heat shock. **G** Partial restoration of *cmyb* signals in *sart3*^*smu471*^ mutants by *mdm4* overexpression (OE). Red arrowheads indicate *cmyb* signals by WISH in the CHT region of 4 dpf larvae. Scale bars: 200 µm.

Previous studies have demonstrated SART3 to promote assembly of U4/U6 snRNPs [32, 50]. Deletion of SART3 reduces functional U4/U6 di-snRNPs which have an essential role in pre-mRNA splicing [32]. To assess *sart3* regulation of HSPC development, we completed AS analysis based on RNA-seq data. We identified 2075 *sart3*-affected AS events including; 1327 skipped exons, 159 retained introns, 145 alternative 3′ splice sites, 81 alternative 5′ splice sites, and 363 mutually exclusive exons (Fig. S7A). To determine whether *sart3*-affected AS events associated with p53 activation, we analyzed genes differentially expressed in the *sart3*^*smu471*^ mutant (5424 genes) with AS (2002 genes) and found 607 genes that overlapped (Fig. 5A). Based on the overlapped genes, we performed Gene Ontology (GO) term enrichment analysis and KEGG pathway analysis using the Metascape database [51] and found ‘mRNA processing’ to be the most enriched pathway (Fig. 5B). The ‘p53 signaling pathway’ was also enriched (Fig. 5B). Clustering analysis of the enriched p53 signaling pathway showed that seven genes (*ptenb*, *adgrg6*, *thbs1b*, *adgrb1a*, *ccnd2a*, *p53*, and *baxa*) were upregulated and six genes (*ddb2*, *tek*, *elmo1*, *mdm4*, *rrm2*, and *syk*) were downregulated (Fig. 5C). Of note, based on replicate multivariate analysis of transcript splicing (rMATS) analysis, *mdm4* in *sart3*^*smu471*^ mutants lacked exon 6 (Fig. S7B), which would result in a truncated *mdm4* transcript (*mdm4-S*) [52]. After analysis of the *mdm4* transcript ratio between the *mdm4* full-length transcript (*mdm4-FL*) and *mdm4-S*, we found that *mdm4-FL* was decreased in *sart3*^*smu471*^ mutants compared with siblings, whereas the *mdm4-S* was increased (Fig. 5D). By monitoring expression of *mdm4-FL* in *sart3* mutants, we found levels to be decreased at 2.5 and 4 dpf (Fig. 5E), which suggested an insufficiency of the correct *mdm4* transcripts during zebrafish development.

MDM4 was known to inhibit the transcription of p53 by binding its transactivation domain [53], and promote p53 protein degradation by interacting with MDM2 [54]. Besides, p53 could regulate itself transcription [55]. Thus, we speculated that elevated p53 expression and enhanced protein activity in *sart3*^*smu471*^ mutants were attributed to AS and downregulation of *mdm4*. To confirm the role of *mdm4* in *sart3*-regulation of HSPC development, we performed a functional assay that restored *mdm4* in *sart3*^*smu471*^ mutants. We found that decreased HSPCs in *sart3*^*smu471*^ mutants could be partially rescued by overexpressing *mdm4* (Fig. 5F, G), suggesting that HSPC deficiency due to *sart3* mutation was, at least in part, a result of *mdm4* insufficiency.

## Discussion

**Fig. 6:**
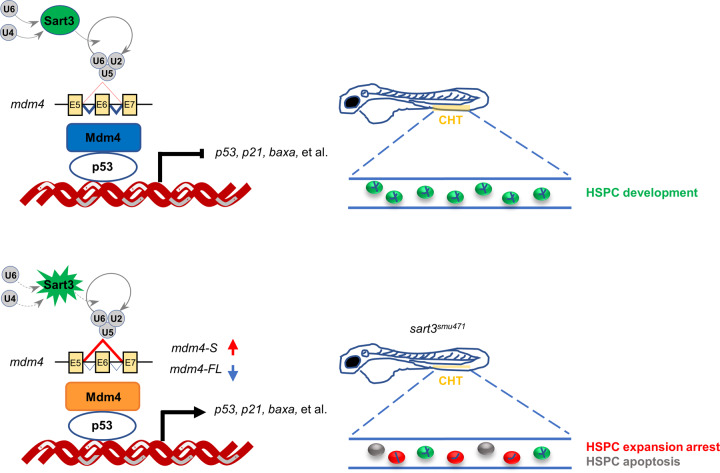
Schematic diagram of Sart3-Mdm4-p53-regulated HSPC development in zebrafish. Schematic diagram of HSPC development regulated by Sart3-Mdm4-p53 pathway in zebrafish. Sart3, which could recycle U4 and U6 snRNPs into spliceosome assembly to affect pre-mRNA splicing [43], promotes HSPC development (current study). Mutation of Sart3 (highlighted in burst) reduces efficient recycling of U4 and U6 snRNPs (broken lines) so that normal splicing of *mdm4* is disrupted. *Mdm4* exon 6 (E6) skipping by *sart3* mutation results in increased truncated *mdm4* transcript (*mdm4-S*) and decreased *mdm4* full-length transcript (*mdm4-FL*). The insufficiency of Mdm4 (orange rectangle) triggers activation of p53 pathway and promotes HSPCs to undergo proliferation arrest and apoptosis in zebrafish early hematopoiesis.

In this study, we mapped the zebrafish *smu471* mutation and identified a *sart3* nonsense mutant that disrupted early HSPC development resulting in definitive hematopoiesis failure. Furthermore, we showed that splicing dysregulation and p53 pathway activation resulted in definitive hematopoiesis failure in *sart3*^*smu471*^ mutants. Overall, these results demonstrate that impairment of *mdm4* by *sart3* mutation activates p53 and its downstream pathway, affecting HSPC proliferation and apoptosis during early hematopoiesis (Fig. 6).

During adult hematopoiesis, mouse *Sart3* has been associated with HPC numbers, survival, and cell cycling, which are likely linked to regulation of *CMYC* [36]. In early hematopoiesis, study of the zebrafish *egy* mutation (another *sart3* zebrafish mutant) found that *sart3* mutation resulted in multi-organ defects including a definitive hematopoiesis defect in which the T cell marker (*rag1*) was absent but primitive erythropoiesis intact [43]. Whether and how developing HSPCs of the *egy* mutant are affected remain unknown. In the present study, we report the *smu471* zebrafish to be a different allele of the *sart3* mutant. The *smu471* mutation exhibited microcephaly and microphthalmia at 4 dpf with death at ~8 dpf (data are not shown). Notably, the *smu471* mutant had definitive hematopoiesis failure identical to the *egy* mutant. Primitive hematopoiesis and definitive HSPC initiation were not disrupted. We attributed definitive hematopoiesis failure to decreased proliferation and increased apoptosis of HSPCs by the *smu471* mutation. Taken together, our results and those of others identify a critical role for *sart3* in various HSPC development and maintenance stages.

We found the ‘spliceosome’ and the ‘p53 signaling pathway’ to be the most enriched pathways in the *sart3*^*smu471*^ mutant by KEGG pathway enrichment analysis of the RNA-seq data. Consistent with our findings, previous microarray analysis of the *egy* mutant showed that half of upregulated genes were snRNP encoding genes and splicing-related factors [43]. Similarly, we also noticed *p53* upregulated in *egy* mutant. These observations suggest that both *egy* and *smu471 sart3* mutation alleles affect hematopoietic development by control of similar molecular pathways.

It is well-known that p53 plays essential roles in cell cycle control and cell death [56, 57]. Further, p53 is highly expressed in HSPCs, wherein it mediates quiescence, self-renewal, and apoptosis [58, 59]. Knock down of *SART3* in hypoxic human U2OS cancer cells significantly decreased p53 protein levels, and p53 could activate *SART3* transcription [37]. However, in whole zebrafish larvae and in endogenous HSPCs, *p53* and its regulated genes were elevated by *sart3* mutation. In addition, downregulation of p53 efficiently restored HSPC numbers in zebrafish. Reduced HSPC levels were due to decreased cell proliferation and increased apoptosis in the *sart3*^*smu471*^ mutant. Our speculation is that *sart3* maintenance of HSPC development is due to p53-dependent inhibition of cellular proliferation and enhanced apoptosis.

*Sart3* is known to be a recycling factor for spliceosome assembly [43]. The ‘spliceosome’ is also the most enriched pathway in the *sart3*^*smu471*^ mutant RNA profile. The *sart3*^*smu471*^ mutant exhibited a transcriptome-wide RNA splicing defect. Several reports suggest that p53 activation may be a consequence of a spliceosome defect [60–62], with our results providing new evidence supporting this hypothesis. It is known that MDM4 could inhibit the transcription of p53 and stimulate the MDM2-mediated protein degradation of p53 [53, 54]. We found *mdm4* to undergo significantly reduced expression along with disrupted AS. *Mdm4* expression was changed by *sart3* mutation, which is consistent with a report demonstrating reduced *mdm4* expression to interfere with the spliceosome [60]. Zebrafish AS of *mdm4* in which exon 6 is skipped in the *sart3*^*smu471*^ mutant is similar to another HSPC deficient zebrafish spliceosome mutant, *bcas2*^−/−^ [62] and to the skipping of exon 6 in human or exon 7 in mouse [63, 64]. A truncated transcript termed *mdm4-S* was previously identified [52]. The conservation of spliceosome-affected *mdm4* and p53 activation across species suggests a general spliceosome-*mdm4*-p53 regulatory pathway in vertebrates.

Moreover, *SART3* alteration may be a favorable index for cancer diagnosis, prognosis, and therapy. For example, *SART3* expression may be a diagnostic and prognostic indicator of melanoma [65]. Antisense oligonucleotides-mediated *MDM4* exon 6 skipping could prove useful as a therapeutic strategy for breast carcinoma, melanoma, and diffuse large B cell lymphoma (DLBCL), by decreasing *MDM4* abundance and reducing the growth of these tumors [63]. Interestingly, we found several DLBCL cases with *SART3* alteration in the cBioPortal database (data are not shown). Thus, whether *SART3* is a candidate prognostic marker or a therapeutic target for DLBCL is worth future investigation. We have shown that human *SART3* mRNA rescues the zebrafish *sart3*^*smu471*^ mutant (Fig. S5B). The *sart3*^*smu471*^ zebrafish could serve as an in vivo model for functional validation of patient-derived mutations as well as a tool for cancer therapeutic approach development.

Taken together, this study describes a novel function for *sart3* in which HSPC proliferation and apoptosis are regulated. Further, we found that AS disruption in the *sart3*^*smu471*^ mutant included effects on the p53 repressor, *mdm4*, which activated p53. Activated p53 was triggered by *mdm4* exon 6 skipping and therefore *sart3* mutation may be a favorable prognostic marker or a potential therapeutic target for some cancers. This study expands understanding of the mechanisms that regulate HSPC development and sheds light on the mechanistic basis and potential therapeutic treatments for *sart3* related disorders.

## Materials and methods

### Zebrafish strains

Zebrafish were raised and maintained in standard conditions [66] with a 14-h light/10-h dark cycle at 28.5 °C. The following zebrafish strains were used; AB, WIK, *Tg*(*cd41:eGFP*) [67], *sart3*^*smu471*^, and *p53*^*M214K*^ [49].

### Genetic mapping and positional cloning

Mapping family generation and positional cloning were performed as previously described [68]. Heterozygous *smu471* [45] were outcrossed to the mapping strain WIK to generate the F1 mapping population, which would be further intercrossed to generate F2 gynogenetic diploid offspring. To verify the mutant phenotype, F2 individuals were subjected to whole mount in situ hybridization (WISH) by *rag1* antisense probe at 5 dpf for genetic mapping. The candidate interval was flanked by two SSLP markers: z20360 and z21082. The 20 candidate genes in this region were all subjected to exon region sequencing in both siblings and *smu471* mutants.

### Generation of *sart3* mutants by CRISPR/Cas9

*sart3*^*(Δ14,+4)*^ mutants were generated using the CRISPR/Cas9 system [69]. The guide RNA (gRNA) targeting sequence was as follows: 5′-GGAGCGAGAGATGGAGTCCG-3′, with the gRNA synthesized using T7 RNA polymerase (EP0111, Thermo Fisher Scientific, San Jose, CA, USA). The mixture of gRNA target (500 ng/µL) and Cas9 protein (600 ng/µL) (M0646M, New England Biolabs, Ipswich, MA, USA) was co-injected into one-cell stage embryos and the mutation efficiency assessed by T7E1 (M0302S, New England Biolabs) digestion. The injected embryos were raised to sexual maturity and screened for a stable F2 heterozygous phenotype of interest.

### Microinjection of mRNAs, plasmids, and MO

The coding sequence of *sart3* was PCR amplified and cloned into the pCS2^+^ vector, then the *sart3* mRNA was synthesized in vitro by mMESSAGE mMACHINE™ SP6 in vitro Transcription Kit (AM1340, Invitrogen, Carlsbad, CA, USA). The coding sequence of *mdm4* was PCR amplified and cloned into the pTol2-*hsp 70* vector. The *p53* MO (5′-GCGCCATTGCTTTGCAAGAATTG-3′) was ordered from Gene Tools as described previously [70]. Microinjection was conducted with 1.5 μg/μL *sart3* mRNA, 70 ng/μL pTol2-*hsp 70: mdm4*, and 1 mM *p53* MO. The construction primers of *sart3* and *mdm4* used in this study are listed in Table S1.

### WISH and genotyping

WISH was performed as described previously [71]. After imaging, the DNA of whole embryo/larva was extracted for genotyping to distinguish *sart3*^*smu471*^ mutants and siblings. PCR was performed to obtain DNA fragments including the point mutation site (forward primer: 5′-GACTGGGCAGATGATGGCGT-3′; reverse primer: 5′-CACCGCATCTGCTCCCAGAGAC-3′), then the PCR products were analyzed by high-resolution melting (HRM) genotyping, with the wild type (WT) and mutant showing one peak and the heterozygote showing two different peaks to initially distinguish those that were heterozygous. Next, we added pre-prepared WT PCR products to the one peak samples for second round HRM analysis, such that the WT showed one peak while the mutant showed two different peaks, as heterozygous.

### Antibody staining

Progenies of the intercrossed *sart3*^*+/smu471*^;*Tg(cd41:eGFP)*^*+/+*^ transgenic zebrafish were fixed and antibody staining was performed as previously described [72]. Primary antibody anti-GFP (1:400, ab6658, Abcam, Cambridge, UK) and secondary antibody Alexa Fluor 488-conjugated anti-goat (1:400, A32814, Invitrogen) were used. Images were captured using a Zeiss LSM800 laser scanning confocal microscope and *cd41:eGFP*^low^ labeled HSPCs were distinguished from *cd41:eGFP*^high^ labeled thrombocytes [46] by the Range Indicator tool of ZEN software according to fluorescence intensities.

### BrdU incorporation and double staining

Progenies of the intercrossed *sart3*^*+/smu471*^;*Tg(cd41:eGFP)*^*+/+*^ transgenic zebrafish were treated with 10 mM BrdU for 3 h at 28.5 °C. After fixation, dehydration, rehydration, and digestion with proteinase K, BrdU labeled embryos were incubated with anti-BrdU (1:50, 111703760001, Roche, Basel, Switzerland) and anti-GFP (1:400, ab6658, Abcam), then incubated with goat Alexa Fluor 555-conjugated anti-mouse (1:400, A31570, Invitrogen) and donkey Alexa Fluor 488-conjugated anti-goat (1:400, A32814, Invitrogen) secondary antibodies for immunofluorescent staining as previously described [73, 74]. The fluorescent images were captured using a Zeiss LSM800 laser scanning confocal microscope with a dual laser channel. According to fluorescence intensities, *cd41:eGFP*^low^ labeled HSPCs were distinguished from *cd41:eGFP*^high^ labeled thrombocytes as mentioned above. Double-positive cells were identified by layer-to-layer examination. Statistical analysis was performed after distinguishing siblings and mutants by HRM genotyping of each sample.

### TUNEL immunostaining and double staining

TUNEL was performed with an In Situ Cell Death Detection Kit TMR red (12156792910, Roche) as described [73, 74]. Initially, the *sart3*^*+/smu471*^;*Tg(cd41:eGFP)*^*+/+*^ transgenic zebrafish intercrossed progenies were fixed, dehydrated, rehydrated, and digested with proteinase K. After incubation with permeabilization solution (acetone:ethanol = 1:2) at −20 °C for 7 min, the embryos were rinsed and stained with the Kit according to the manufacturer’s instructions. Imaging, quantification, and statistical analysis were carried out as described above.

### RNA-seq and RT-qPCR

Total RNA of desired stage embryos/larvae or sorted-HSPCs was extracted using Trizol reagent (15596026, Invitrogen) according to the manufacturer’s instructions. For RNA-seq, the sequencing libraries were generated by NEBNext® UltraTM RNA Library Prep Kit for Illumina® (E7770, New England Biolabs). For RT-qPCR, cDNA was prepared with HiScript Q RT SuperMix for qPCR (+gDNA wiper) (R323-01, Vazyme, Nanjing, China). The RT-qPCR was performed using PowerUp™ SYBR^®^ Green Master Mix (4472908, Thermo Fisher Scientific) in a LightCycler 96 system (Roche). The RT-qPCR primers used in this study are listed in Table S2.

### AS analysis and detection

For AS events analysis, rMATS software was used [75]. For *mdm4* AS detection, cDNA of *sart3*^*smu471*^ mutants and their siblings was prepared as above-mentioned, then *mdm4* AS was visualized by PCR using cDNA template. AS detection primers used in this study are listed in Table S3.

### Western blot

To obtain zebrafish protein, we distinguished 4-dpf *sart3*^*smu471*^ mutants and their siblings based on the microcephaly and microphthalmia phenotype of the *sart3*^*smu471*^ mutant, then extracted total protein for western blot as previously described [76]. Mouse monoclonal antibody anti-p53 (1:200, ab77813, Abcam) [77, 78] and rabbit monoclonal antibody anti-GAPDH (1:1000, #2118, Cell Signaling Technology, Danvers, MA, USA) were used as primary antibodies.

### FACS analysis

The *sart3*^*+/smu471*^;*Tg(cd41:eGFP)*^*+/+*^ zebrafish were intercrossed and 4-dpf *sart3*^*smu471*^ mutants as well their siblings were distinguished as mentioned above. The FACS experiment was performed as described previously [79]. As reported, *cd41:eGFP*^low^ labeled cells were recognized as HSPCs [46], and were, respectively, analyzed or sorted from *sart3*^*smu471*^ mutants and siblings using a MoFlo™ XDP (Beckman Coulter, Brea, CA, USA).

### Statistical analysis

Data were recorded and analyzed using GraphPad Prism 7. Categorical variables were analyzed by Fisher’s exact test. Comparisons between two groups were analyzed by two-tailed Student’s *t*-test and nonparametric test (Mann–Whitney *U* test), based on normal distribution data or not, respectively. Data were presented as means ± standard deviation (SD). *P* < 0.05 was considered statistically significant.

## Supplementary information

Zhao et al, supplementary

## Data Availability

The RNA-seq data was submitted to the NCBI GEO repository with the accession number GSE182560.
