# Supplementary material for: The spliceosome factor sart3 regulates hematopoietic stem/progenitor cell development in zebrafish through the p53 pathway
Source: Cell Death Dis. 2021 Oct 5;12(10):906. doi: 10.1038/s41419-021-04215-4 (PMC8492694; doi:10.1038/s41419-021-04215-4)
Supplement: Supplementary file 1 — Zhao et al, supplementary [file 41419_2021_4215_MOESM1_ESM.docx]

**Supplementary Figures and Legends**


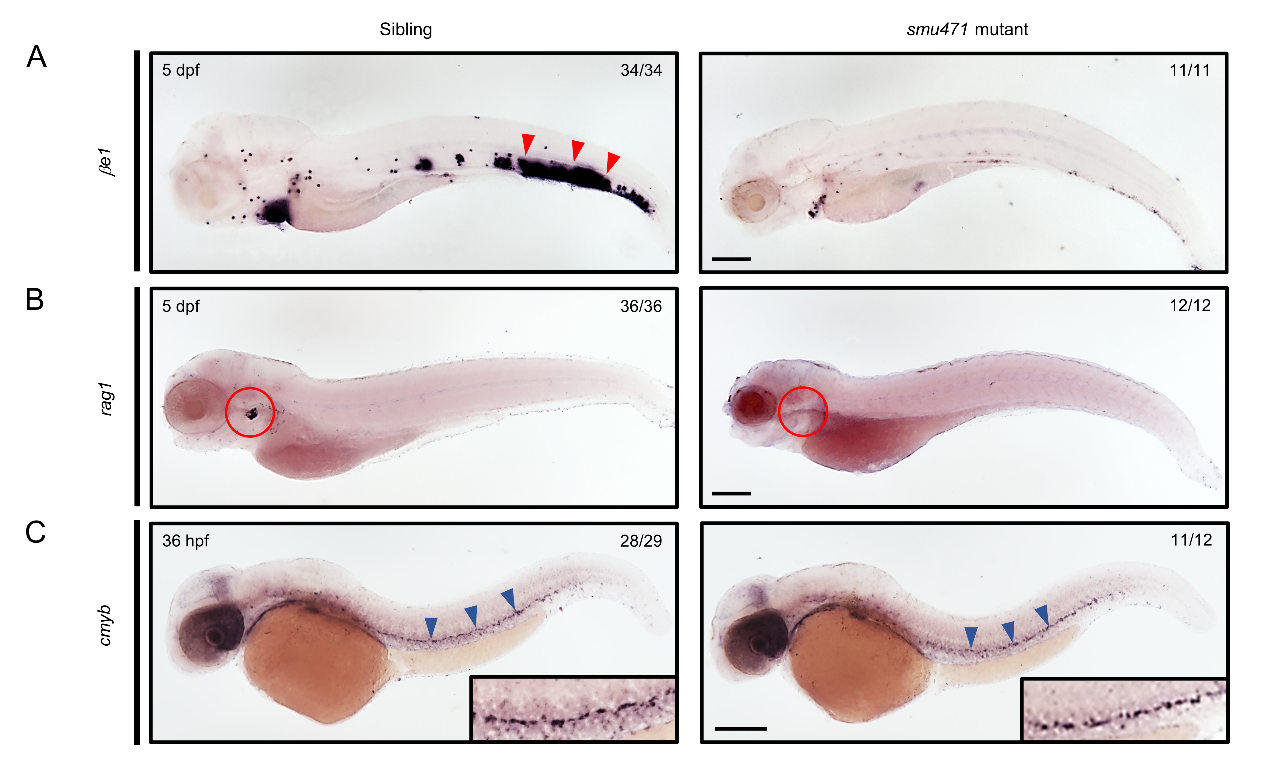


**Figure S1. Definitive hematopoiesis is defective but HSPC initiation is normal in zebrafish *smu471* mutants.**

(A, B) WISH results show definitive hematopoiesis to be significantly defective in *smu471* mutants. The erythroid maker *βe1* (red arrowheads indicate signals in the CHT region) (A) and lymphoid maker *rag1* (red circles indicate signals in the thymus region) (B) were decreased in *smu471* mutants at 5 dpf. (C) WISH results demonstrate HSPC initiation to be unaffected in *smu471* mutants. The HSPC marker *cmyb* (blue arrowheads indicate signals in the VDA region) was normal at 36 hpf and similar for *smu471* mutants and their siblings. Scale bars: 200 µm.


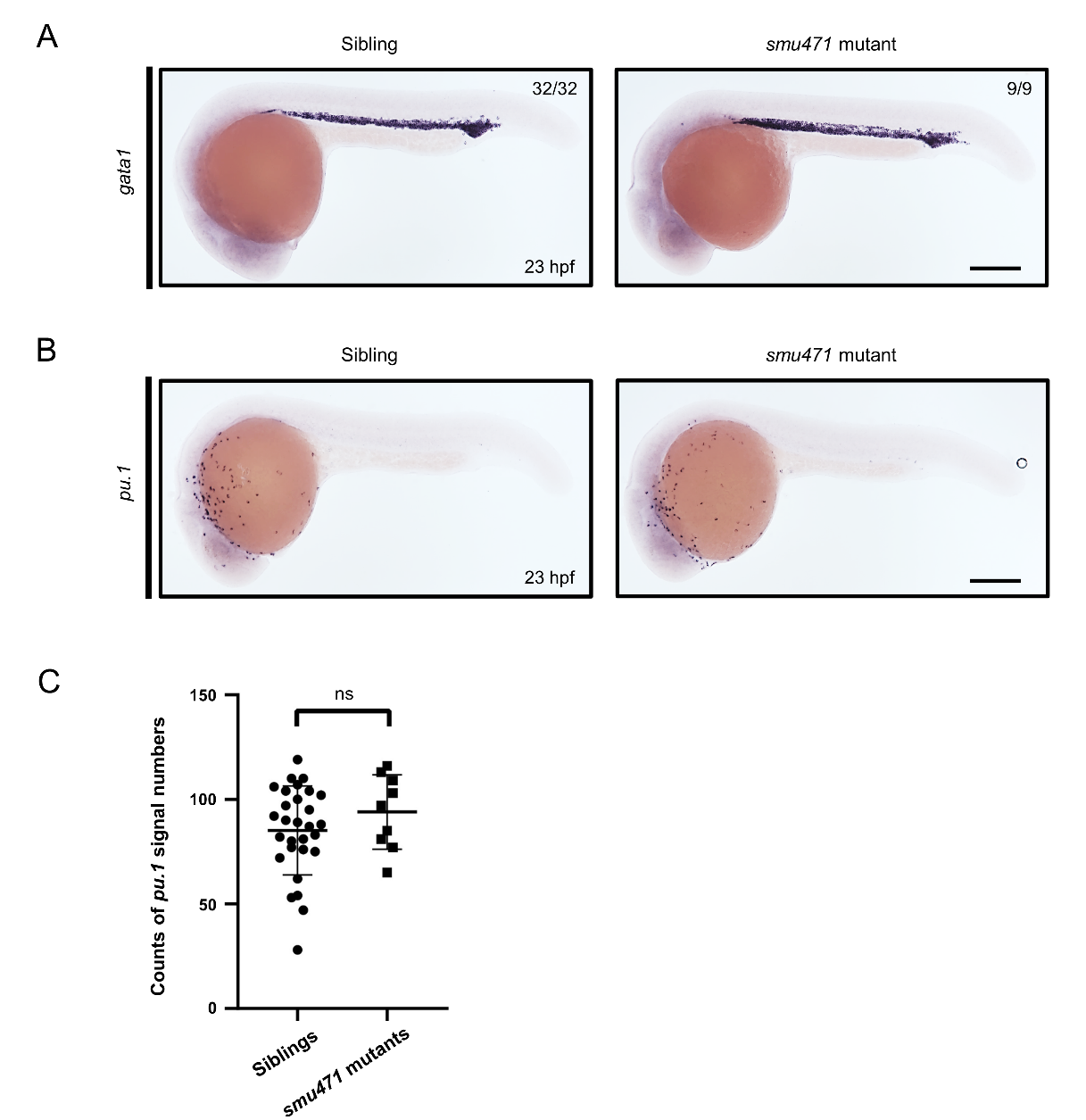


**Figure S2. Primitive hematopoiesis is unaffected in zebrafish *smu471* mutants.**

(A, B) WISH results demonstrate primitive hematopoiesis to be intact in *smu71* mutants and siblings. The primitive erythrocyte progenitor marker *gata1* (A) and myeloid progenitor marker *pu.1* (B) were unaffected at 23 hpf in *smu71* mutants and siblings. (C) Quantifications of the *pu.1^+^* signals in (B) (Student’s *t-*test, means ± SD; siblings, n = 29; *smu471* mutants, n = 9; ns: not significant). Scale bars: 200 µm.


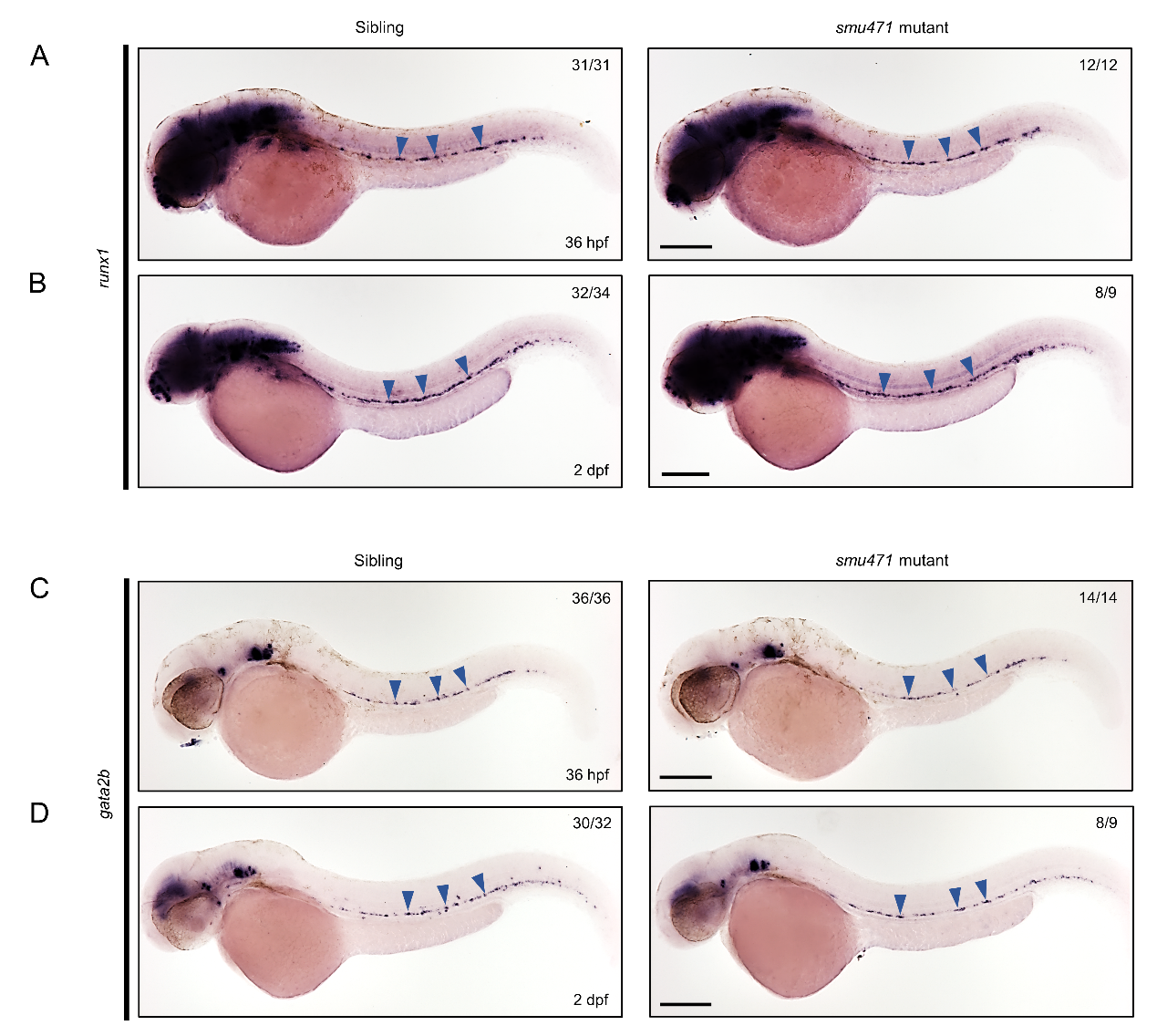


**Figure S3. HSPCs are unaffected in *smu471* mutants at 36 hpf and 2 dpf.**

(A, B) The HSPC marker *runx1* was normal at 36 hpf (A) and 2 dpf (B) in *smu471* mutants and similar to siblings. (C, D) The HSPC marker *gata2b* was normal at 36 hpf (C) and 2 dpf (D) in *smu471* mutants and similar to siblings. Blue arrowheads indicate signals in the VDA region. Scale bars: 200 µm.


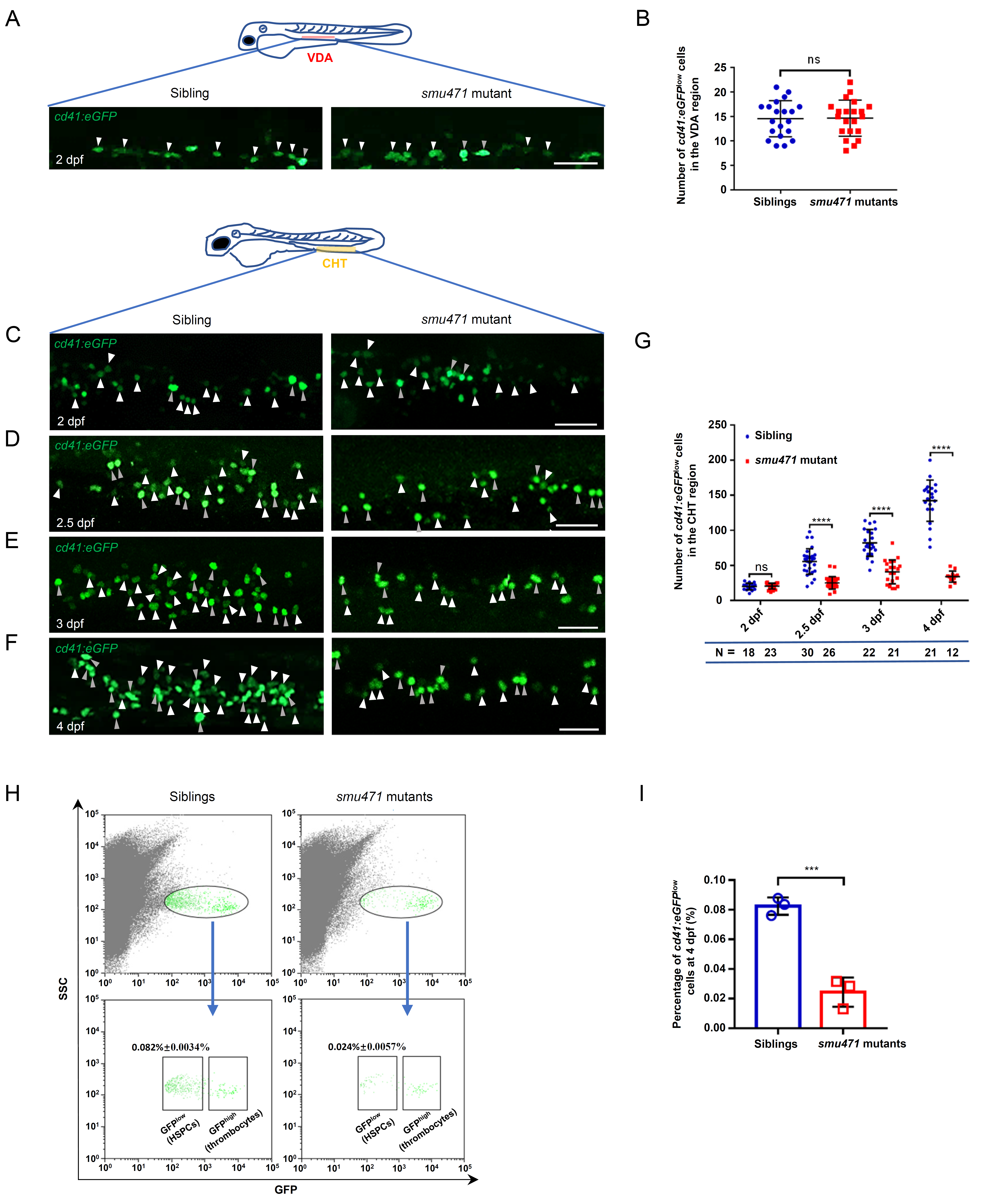


**Figure S4. HSPCs are decreased in *smu471* mutants from 2.5 dpf onward.**

(A) Fluorescent images show the expression of *cd41:eGFP^+^* cells in the VDA region at 2 dpf in *smu471* mutants and their siblings. The white arrowheads indicate the *cd41:eGFP*^low^ labelled HSPCs (calculated for quantification), and the grey arrowheads indicate the *cd41:eGFP*^high^ labelled thrombocytes (excluded from the quantification). Scale bars: 50 µm. (B) Quantification of *cd41:eGFP*^low^ labelled cells in the VDA region. Student’s *t*-test, mean±SD; siblings, n = 20; *smu471* mutants, n = 21, ns: not significant. (C-G) Fluorescent images show the expression of *cd41:eGFP^+^* cells in the CHT region at 2 dpf (C), 2.5 dpf (D), 3 dpf (E), and 4 dpf (F) in *smu471* mutants and their siblings. Scale bars: 50 µm. (G) Quantification of *cd41:eGFP*^low^ labelled HSPCs in the CHT region. Data are presented as mean±SD. ****P<0.0001, ns: not significant, Student’s *t*-test. N indicates the sample number of each group. (H) FACS plots show the proportion of *cd41:eGFP*^low^ cells in siblings and *smu471* mutants at 4 dpf. (I) Quantification of *cd41:eGFP*^low^ labelled cell population in (H). Data are presented as mean±SD of three independent experiments. ***P<0.001, Student’s *t*-test.


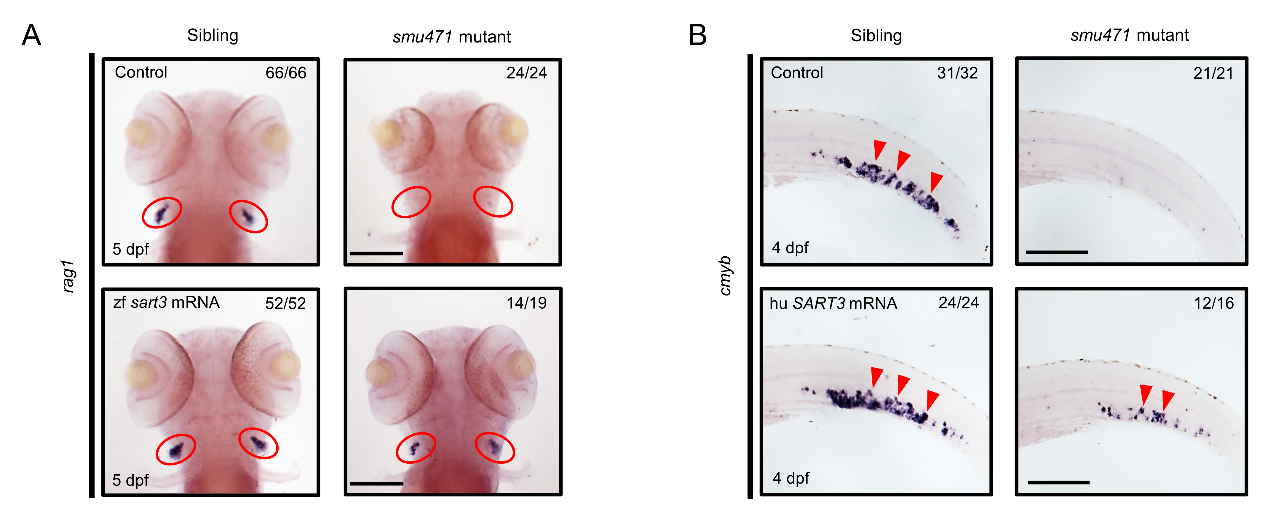


**Figure S5. *sart3* is the causative gene of the zebrafish *smu471* mutation.**

(A) Restoration of *rag1* expression by zf *sart3* mRNA in *smu471* mutants. WISH of *rag1* was performed with 5-dpf siblings and *smu471* mutant larvae with or without zf *sart3* mRNA injection. Red circles indicate WISH signals of *rag1* in the thymus region. (B) Restoration of *cmyb* expression by overexpression of human *SART3* mRNA (hu *SART3* mRNA) in *smu471* zebrafish mutants. WISH of *cmyb* was performed with 4-dpf siblings and *smu471* mutant larvae with or without mRNA injection. Red arrowheads indicate WISH signals of *cmyb* in the CHT region. Scale bars: 200 µm.


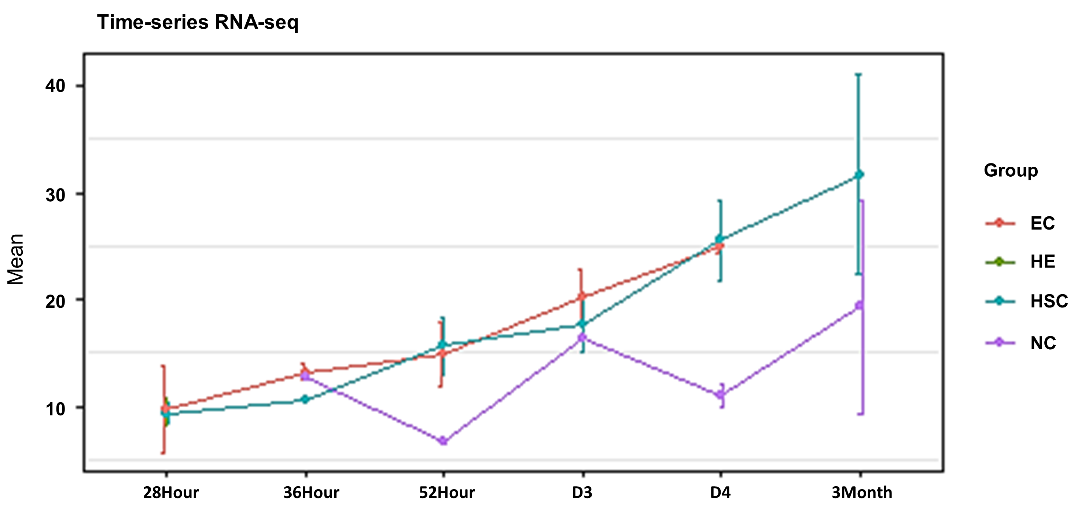


**Figure S6. Dynamic expression of *sart3* in zebrafish HSCs.**

Gradually increased HSC expression of *sart3* in the zebrafish CHT region based on published time-series RNA-seq data (the figure was generated from the website <https://www.picb.ac.cn/hanlab/ichtatlas/Home/>) [47]. EC: endothelial cells (red line); HE: hemogenic endothelium (green line); HSC: hematopoietic stem cells (blue line); NC: non-endothelial/non-hematopoietic cells (purple line).


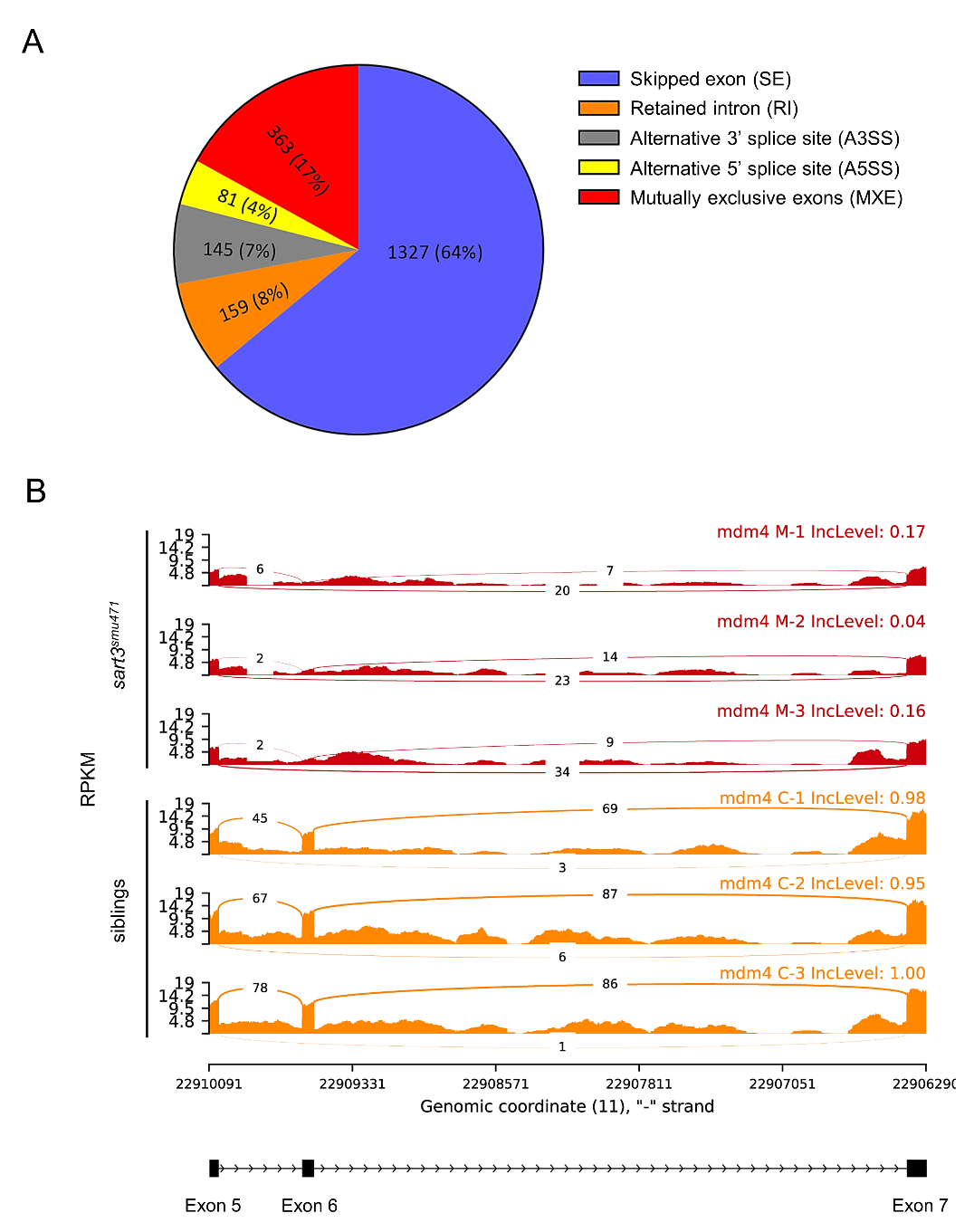


**Figure S7. *sart3* mutation triggers global alternative splicing and *mdm4* exon 6 skipping.**

(A) All alternative splicing events in *sart3^smu471^* mutants and every type of alternative splicing event, number, and percentage are presented. (B) *mdm4* exon 6 skipping in *sart3^smu471^* mutants. Sashimi plot to visualize the RNA-seq mapping reads at the exon 5–7 loci of *sart3* in *sart3^smu471^* mutants and their siblings.

**Supplementary Tables**

**Table S1. The list of construction primers.**

| **Gene** | **Primer sequences (5′-3′)** |
| --- | --- |
| *hsp-mdm4*  zf *sart3*  hu *SART3* | GGGACCGGTATGCAATGAAGGTTTTGATGACCT  CGCGGATCCATGACCTCATTGGCATCGTCATCT  CGCGGATCCGAAGTTCTCAACATGGCGGCG  CCGGAATTCGGGCTTCATAAGGACACAGCATGG  GCTACTTGTTCTTTTTGCAGATGGCGACTGCGGCCGAAAC  CCTTGAATTCGAATCGATGGTCACTTTCTCAGAAACAGCTTGGC |

**Table S2. The list of RT-qPCR primers.**

| **Gene** | **Primer sequences (5′-3′)** |
| --- | --- |
| *p53*  *p21*  *ccng1*  *baxa*  *mdm2*  *gadd45*  *casp8*  *mdm4* | GGGACCAAACGTAAATCTTCTTC  CTCACCTGCAGGGTAAAGATC  CCAACATCACAGATTTCTACCA  TTTCTGTCAATAACGCTGCT  CTTTCACTAAACTAAAGCCTTCCCT  CATTTAAACTCTGCTGCAGACC  TCATCAAGGCTATTTCAACCAG  GTTCCCTGATCCAGTTAATGAC  AGACTCTCGCTCATCTACCTC  ATACCTACATCCGAGTTGCTG  CTCGGTGATTAAGGCTCTGG  GTTATCAGGGTCCACATTGAGG  GTGTCTCAGCCTATAGAAAGATGC  AAGTAGATCTTCCCAGCTTTGCC  CTCCAAGTGAAGATCCTGGTC  CCATCTGAGGAGTCTTCATCTGG |

**Table S3. The list of AS primers.**

| **Gene** | **Primer sequences (5′-3′)** |
| --- | --- |
| *mdm4* exon6 skip | GAGATGTTGAAGAGGAATTTGGT  CCATCTGAGGAGTCTTCATCTGG |
